# Supplementary material for: Synaptic plasticity in self-powered artificial striate cortex for binocular orientation selectivity
Source: Nat Commun. 2022 Sep 23;13:5585. doi: 10.1038/s41467-022-33393-8 (PMC9508249; doi:10.1038/s41467-022-33393-8)
Supplement: Supplementary file 1 — Supplementary Information [file 41467_2022_33393_MOESM1_ESM.pdf]

Supplementary Information

Synaptic plasticity in self-powered artificial striate cortex for binocular orientation selectivity

Yanyun Ren[1,2], Xiaobo Bu[2], Ming Wang[3], Yue Gong[4], Junjie Wang[4], Yuyang Yang[4], Guijun Li[3], Meng Zhang[4], Ye Zhou[2], and Su-Ting Han[4*]

[1]Institute for Microscale Optoeletronics, Shenzhen University, Shenzhen 518060, P. R. China

[2]Institute for Advanced Study, Shenzhen University, Shenzhen 518060, P. R. China

[3]Key Laboratory of Optoelectronic Devices and Systems of Ministry of Education and Guangdong Province, College of Physics and Optoelectronic Engineering, Shenzhen University, Shenzhen 518060, P. R. China

[4]College of Electronics and Information Engineering, Shenzhen University, Shenzhen 518060, P. R. China

*Correspondence and requests for materials should be addressed to S.-T.H. (e-mail: sutinghan@szu.edu.cn)

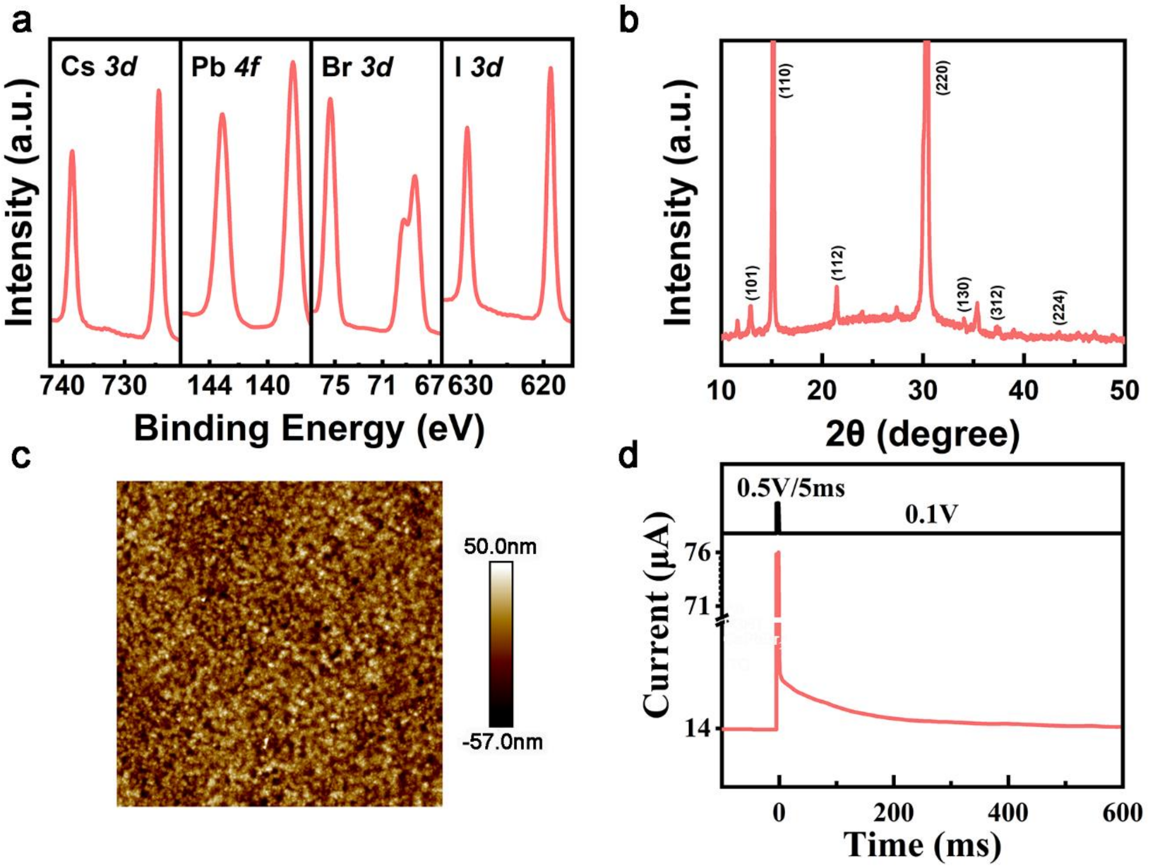

Supplementary Figure 1. Characteristics of memristor. a, High-resolution XPS spectra of the samples at Cs *3d*, Pb *4f*, Br *3d* and I *3d* regions of $CsPbBr_2I$ perovskite film. **b,** XRD pattern of as-fabricated $CsPbBr_2I$ perovskite film. **c,** The AFM image of $CsPbBr_2I$ perovskite film (scan size:20μm× 20μm). **d,** The EPSC behavior of memristor.

The chemical bonding state of perovskite thin film was investigated by X-ray photoelectron spectroscopy (XPS). Figure S1a shows that, the common signature peaks of Cs *3d*, Pb *4f*, Br *3d* and I *3d* can be observed from the $CsPbBr_2I$ film. Figure S1b shows the X-ray diffraction (XRD) pattern of $CsPbBr_2I$ perovskite film, except the diffraction peaks from substrate, the peaks at 12.94, 15.08, 21.44, 30.28, 34.04, 37.34, 43.52 are assigned to the (101), (110), (112), (220), (130), (312), (224) planes of the orthorhombic phase (ICDD: 01-02707929). It indicates that the pure $CsPbBr_2I$ film of orthorhombic phase has formed at room temperature. The atomic force microscopy (AFM) to confirm the surface morphology of perovskite films in memristor, as shown in Figure S1c. The roughness of $CsPbBr_2I$ film in artificial synapse is about 11.8nm with a peak-to-peak distance of 5.1nm, evidencing the high quality of perovskite film used in this work. In order to confirm the device decay behavior, the excitatory postsynaptic current (EPSC) behavior of our memristor has shown in Figure S1d. The decay time before the current relax back to initial state from peak value attend 200ms.

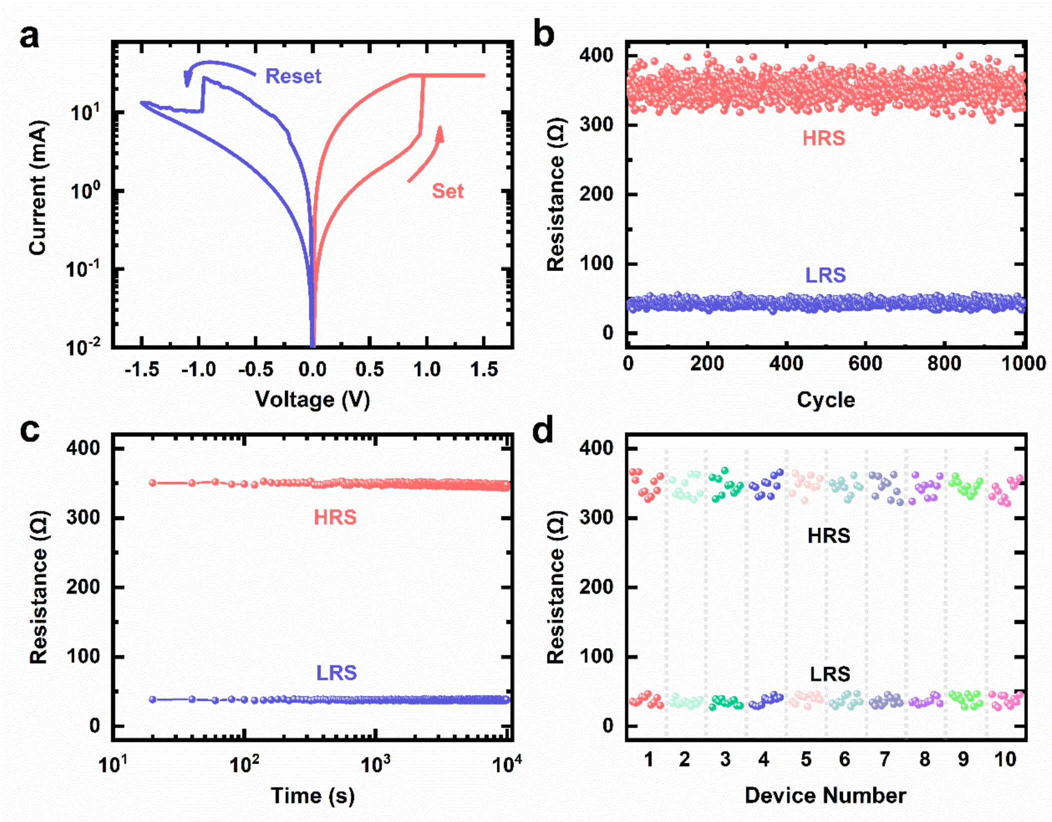

Supplementary Figure 2. a, Resistive switching of memristor with complicance current of 30mA. **b,** The endurance characteristic of the memristor. **c,** The retention characteristic of the memristor. **d,** the HRS and LRS distribution during consecutive 10 cycles from 10 randomly selected memristors.

We performed typical *I-V* characteristics of perovskite memristor under the voltage sweep from 0 to +1.5 V to 0 V to -1.5 V to 0 V with the compliance current (I_{cc}) of 30 mA, as shown in Figure S2a. The memristor shows typical bipolar resistive switching behavior. During the positive scan (SET operation), the memristor initially exhibited a high resistance of 372 Ω with the reading bias of 0.1 V. As the voltage increased to 0.94 V (set voltage), the memristor was abruptly switched to low resistance state (LRS) of 34 Ω with the current reaching the I_{cc}. When the negative voltage applied on TE, the current suddenly decreased at -0.95 V (reset voltage) and device was transited from LRS to HRS. The endurance characteristics is shown in Figure S2b. The resistance states show no degradation with acceptable variation in consecutive 1000 cycles. The HRS and LRS fluctuate in the range of [307 Ω 401 Ω] and [32 Ω 56 Ω], respectively with the switching window of ~8.1 which is comparable to the previous reported perovskite memristor. The Figure S2c shows the retention characteristics of the memristor. The test pulse was set to 0.1V/5ms and the pulse interval was set to 20s. Both HRS and LRS can be well preserved up to 10^4 s, verifying that memristor could retain the conductance state in the long-term. In addition, 100 cycles characterizations have been implemented on 10 randomly selected memristors, and the spatio-temporal distribution of HRS and LRS showed no obvious fluctuation which demonstrate the great C2C and D2D uniformity of the memristor. The variations of HRS and LRS, described as σ/μ (σ is the standard deviation and μ is the mean value), in the endurance, retention and mulit-device tests are calculated and provided in Table S1.

Supplementary Table 1. The mean values, standard deviations and variations of HRS and LRS in the endurance, retention and mulit-device tests.

Parameter	Endurance		Retention		Multi-Device	
	HRS	LRS	HRS	LRS	HRS	LRS
Mean Value	353.11 Ω	43.88 Ω	347.89 Ω	37.99 Ω	344.77 Ω	36.71 Ω
Standard Deviation	15.28 Ω	4.05 Ω	2.11 Ω	0. 56 Ω	12.23 Ω	5.83 Ω
Variation	0.043	0.093	0.006	0.015	0.035	0.159

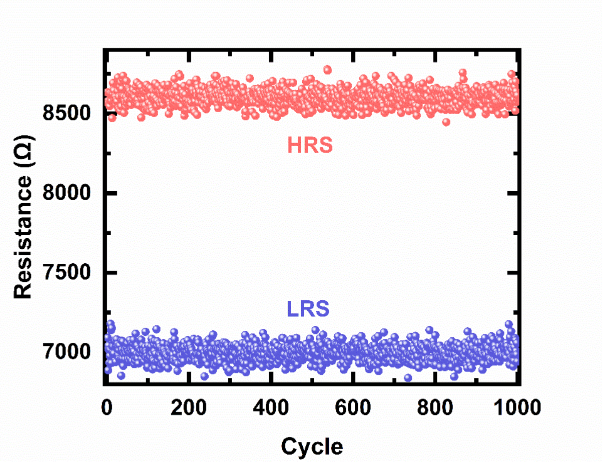

Supplementary Figure 3. The endurance characteristic of the memristor in the pulse-based test.

We implemented the pulse-based endurance test, as shown in Fig. S3. Under the alternate positive 0.5V/5ms and negative -0.5V/5ms pulses, the device shows potentiation and depression effects and the resistance states show almost no degradation in the 1000 cycles.

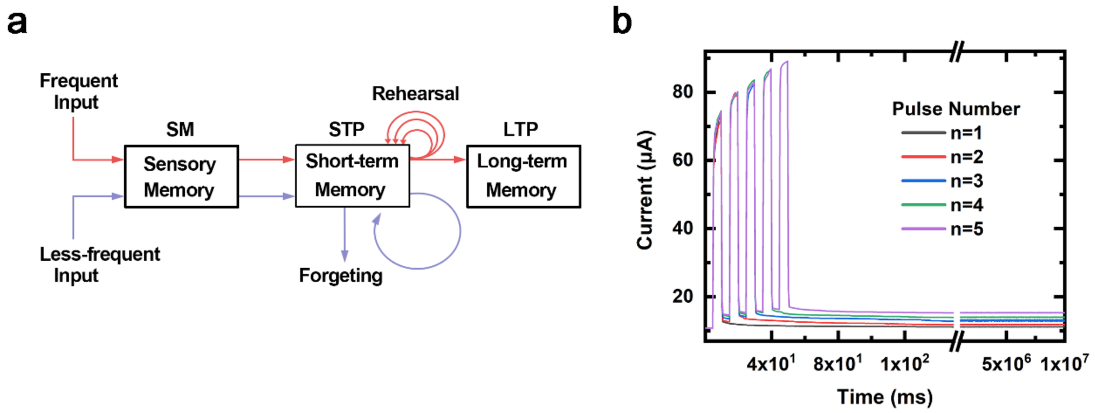

Supplementary Figure 4. a, The biological memory model proposed by Atkinson and Shiffrin. **b,** The memristor current responses to pulse train stimulations with different pulse numbers.

In the psychological model of biology memory system, the perceptive information is firstly stored for a very short period of time in the sensory memory, and then the information can be transformed from short-term memory (STM) to long-term memory (LTM) through a rehearsal process, corresponding to the transition from short-term dynamics to long-term dynamics in neuroscience. To mimic this memory process, we monitored the current states by application of pulse trains ranging from one single pulse to five pulses where the pulse amplitude and width are set to 0.5V and 5ms, respectively. The conductance of memristor decays to original state of 110 μS after application of one single pulse but can be stabilized at 150 μS for 10^4 s after application of five pulses, indicating the transition from short-term dynamics to long-term dynamics.

Non-Linearity

The potentiation and depression in pulse experiment can be fitted as following equations, respectively:

$$\begin{cases} G_{i+1} = G_i + \Delta G_p = G_i + \alpha_p e^{-\beta_p \frac{G_i - G_{min}}{G_{max} - G_{min}}} & \text{LTP} \\ G_{i+1} = G_i + \Delta G_d = G_i - \alpha_d e^{-\beta_d \frac{G_{max} - G_i}{G_{max} - G_{min}}} & \text{LTD} \end{cases} \quad (1)$$

where the G_i and G_{i+1} are the conductance of the ith and $(i+1)^{th}$ pulses, G_{max} and G_{min} are the maximum and minimum conductance of potentiation/depression values, α_p and α_d are the conductance differences between two pulses in the potentiation and depression, β_p and β_d are the non-linearity (NL) of the potentiation and depression, represents the curvatures of the experimental curves. The fitting results of Figure 2e are summarized in Table S2.

Supplementary Table 2. The fitting results of NL from pulse experimental data.

G_{min}	G_{max}	LTP		LTD	
		α_p	β_p	α_d	β_d
50 μS	450 μS	24.72 μS	3.00	49.72 μS	3.98

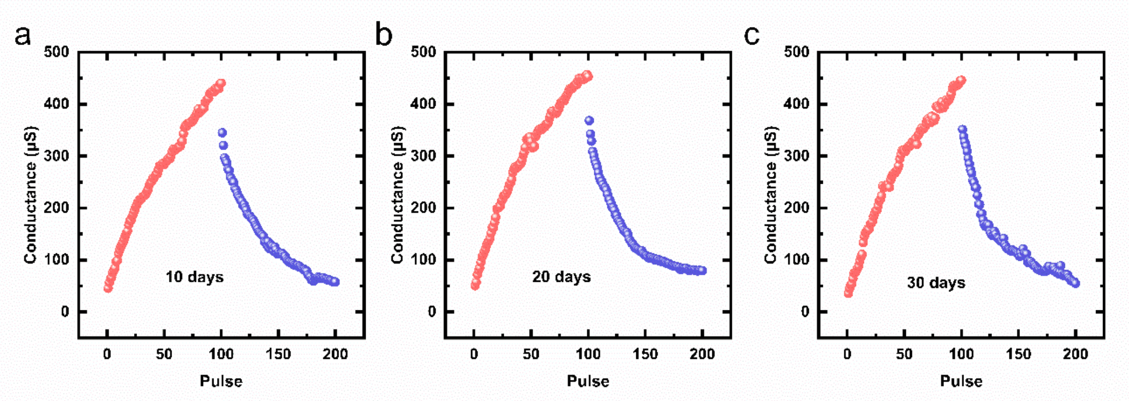

Supplementary Figure 5. The obtained conductance potentiation and depression after **a,** 10 days **b,** 20 days **c,** 30 days.

In order to evaluate the life time of our memristor, we carry out the potentiation and depression measurements with respect to the time eclipse (Figure S5). The memristors were applied with 100 consecutively positive pulses with amplitude of 0.5 V and duration of 5 ms followed by 100 consecutively negative pulses with amplitude of -0.5 V and duration of 5 ms. Thanks to the encapsulation by glass and edge sealed by optical adhesive, the LTP and LTD characteristics of memristor shows no obvious degradation after 10, 20 and 30 days.

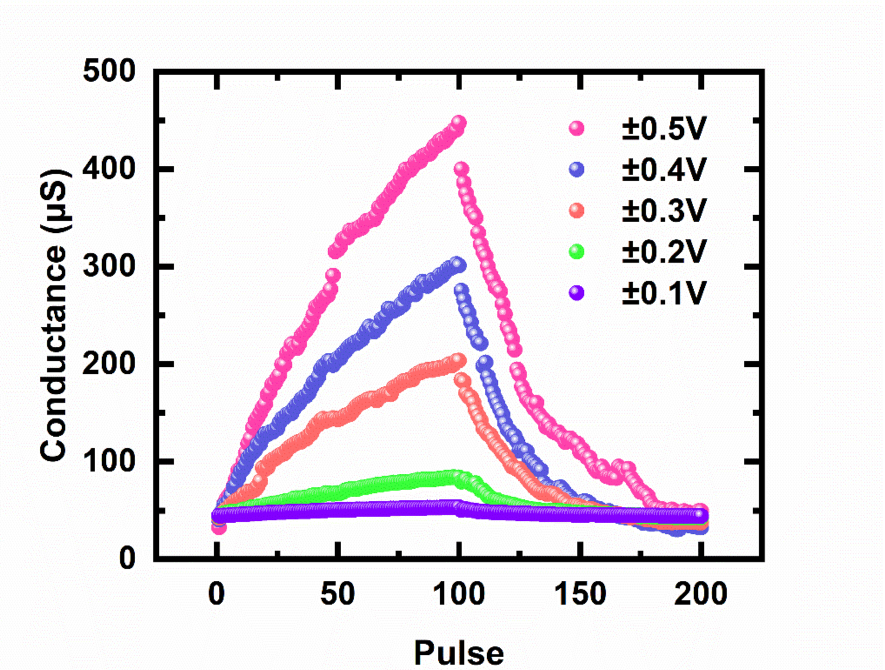

Supplementary Figure 6. LTP and LTD characteristics of memristor by applying various pulse voltages of ±0.5V, ±0.4V, ±0.3V, ±0.2V and ±0.1V, and all the pulse width is set to 0.5ms.

In order to clarify the memristor input voltage range, we carried out the consecutive pulse tests with different pulse amplitudes and fixed pulse width, as shown in Figure S6. It can be seen that the memristor can be modulated by pulse amplitude of ±0.5V, ±0.4V and 0.3V, while the pulse of ±0.2V and ±0.1V cannot trigger the memristor.

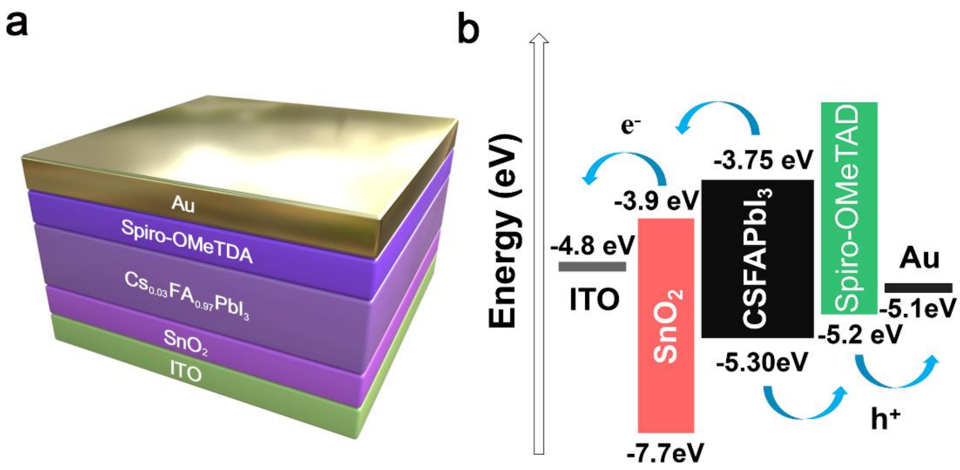

Supplementary Figure 7. The schematic illustration and energy level of perovskite-based solar cell.

The Figure S7a shows the solar cell configuration used in this study with a structure of Au/Spiro-OMeTAD/$Cs_{0.03}FA_{0.97}PbI_3/SnO_2$/ITO. Figure S7b displays the energy level diagram of our solar cell. All energies are referenced to the vacuum energy. A great charge transfer efficiency can be achieved thanks to the matched energy level of each functional film.

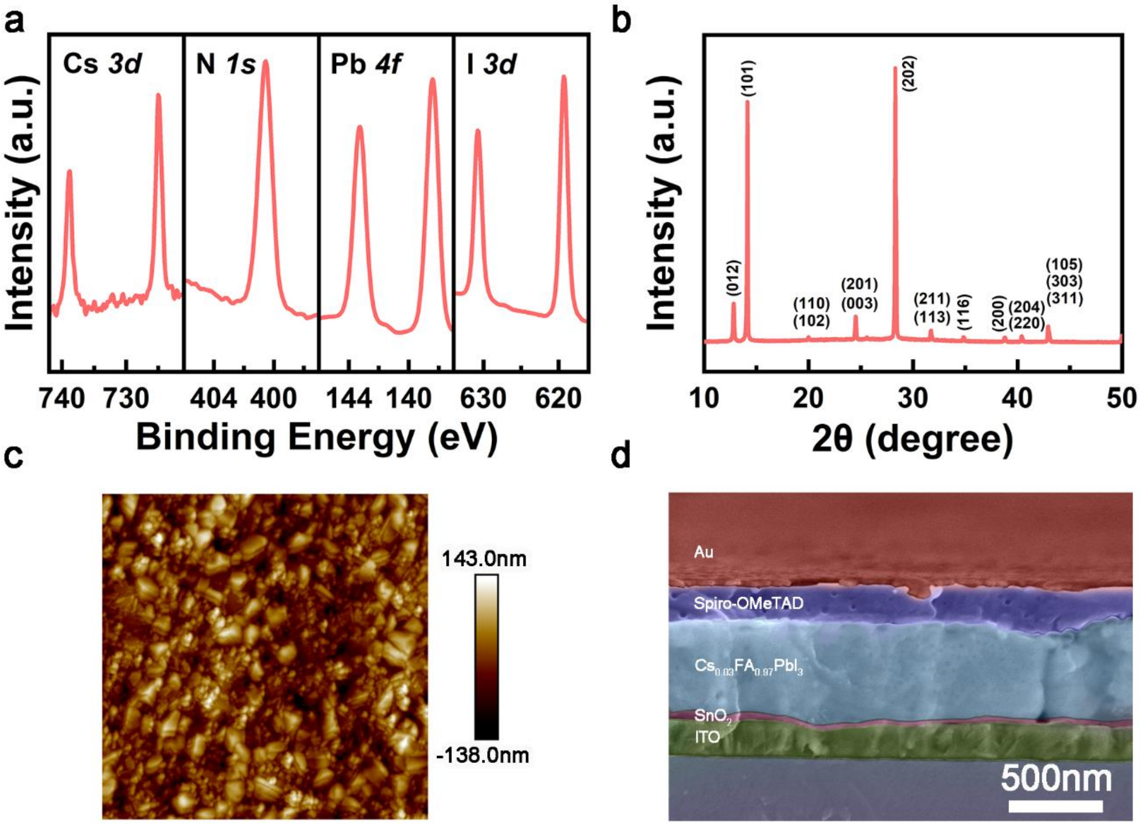

Supplementary Figure 8. Characteristics of Solar cell. a, High-resolution XPS spectra of the samples at Cs *3d*, N *1s*, Pb *4f* and I *3d* regions of $CsFAPbI_3$ perovskite film. **b,** XRD pattern of as-fabricated $CsFAPbI_3$ perovskite film. **c,** The AFM image of $CsFAPbI_3$ perovskite film (scan size: 20μm×20μm). **d,** The SEM image of solar cell (scale bar: 500nm).

As shown in Figure S8a, the clearly Cs *3d*, Pb *4f*, Br *3d* and I *3d* peaks were observed from the $CsFAPbI_3$ film, indicating a great quality of film fabrication. In Figure S8b, the diffraction peaks of (100), (110) and (200) could all indexed to the black phase of $CsFAPbI_3$, which is consistent with the previous studies. From the AFM characteristic, as shown in Figure S8c, the roughness of $CsFAPbI_3$ film is 32.9nm and the peak-to-peak distance is 1.9nm. Figure S8d shows the cross sectional SEM image of solar cell, it is clearly that the perovskite film is inserted at the interface between SnO_2 and Spiro-OMeTAD with a thickness of 500nm.

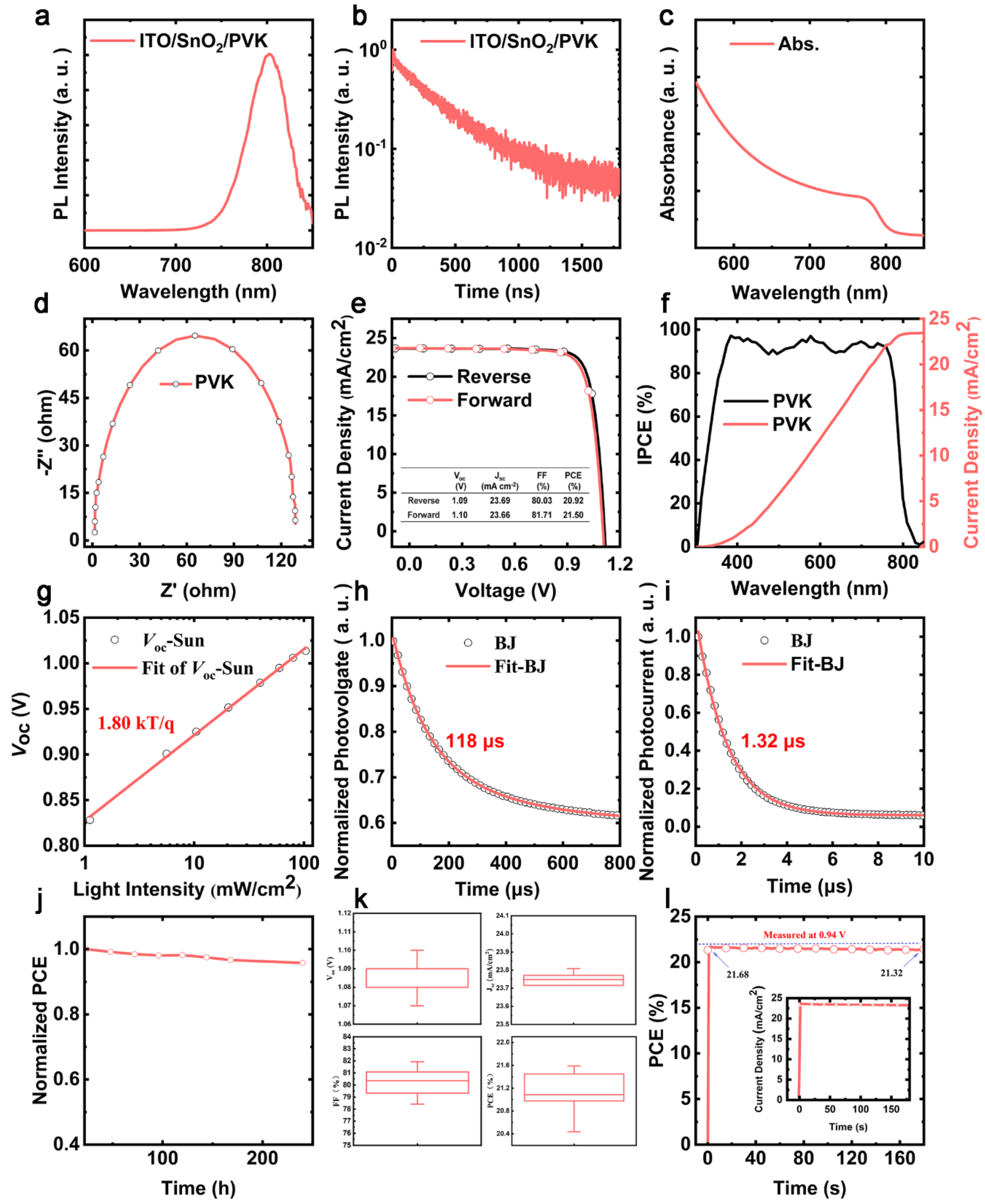

Supplementary Figure 9. Characteristics of solar cell. a, SSPL spectra and **b,** TRPL spectra of target perovskite/SnO_2/ITO device. **c,** The UV-vis spectra of perovskite solution. **d,** ESI spectra of our solar cell. **e,** The J-V curve of the target solar cell, which were measured by reverse scan (1.2V to -0.1V) and forward scan (-0.1 to 1.2V) with a scanning rate of 0.02V/s. **f,** IPCE spectra and integrated J_{sc} of target solar cell. **g,** The V_{oc} versus light intensity for the target solar cell. **h,** The transient photovoltage and **i,** the transient photocurrent decay curves of the target solar cell. **j,** Long-term stability of target solar cell. **k,** Statistics of photovoltaic parameters of target solar cells (10 devices). **l,** Steady-state photocurrents of 180 s at bias voltages of 0.94 V at the maximum power output.

In order to investigate the carrier separation, transport and suppressed trap-assisted non-radiative recombination, the steady-state photoluminescence (SSPL) spectroscopy and time-resolved

photoluminescence (TRPL) spectroscopy were performed as presented in Figure S9a-b. The SSPL peak locates at 803nm. For TRPL spectrum, a biexponential equation $I(t) = I_0 + A_1 exp(-t/\tau_1) + A_2 exp(-t/\tau_2)$ was used to fit the experimental curve, where A_1/A_2 and τ_1/τ_2 are amplitudes and time constant of fast/slow decay process. The τ_1 and τ_2 of our solar cell are 104 ns and 406 ns, indicating a longer carrier lifetime as well as higher charge extraction and transport in the perovskite layer. The Figure S9c shows UV-vis absorption spectrum of perovskite solution where a absorption edge is located at 800nm. The wider absorption spectrum and higher absorption peak are very helpful to the promote photoelectric conversion efficiency. In addition, the electrochemical impedance spectroscopy (EIS) was used to explore the charge transfer process (Figure S9d). The charge transfer resistance (R_{CT}) is composed of the interface resistances between the carrier selective layer and the perovskite layer, as well as the resistance between the electrode and carrier selective layer. Based on the equivalent circuit of Nyquist theorem, the fitting R_{CT} of 132.4Ω means a promoted charge transfer at the interface and reduced interfacial potential barriers. The J-V curve of perovskite solar cell is shown in Figure S9e, where current density of 23.72mA/cm^2 has been achieved. The incident photon to current conversion efficiencies (IPCE) of ~92% and short-circuit photocurrent density (J_{sc}) of 23 mA/cm^2 is obtained (Figure S9f). The light-intensity-dependent open circuit voltage (V_{oc}), transient photovoltage (TPV), transient photocurrent (TPC) were characterized as shown in Figure S9g-i. Following a linear relationship between V_{oc} and light intensity, the ideality factor value of 0.99 indicates that the interfacial non-radiative recombination is low. Besides, the V_{oc} can be enhanced from 0.83 V to 1.01 V by adjusting light intensity, ensuring the next-stage integration with memristor with 0.5 V operation voltage. We evaluated the TPC and TPV of our solar cell, the photocurrent decay lifetime and the photovoltage decay lifetime are 1.32 μs and 118 μs, which verifies the low non-radiative recombination. The solar cell also demonstrated a good device stability, retaining over 96% of its maximum efficiency under continuous simulated solar illumination for 240 hours (Figure S9j). Figure S9k shows the statistics of the photovoltaic parameters of the target solar cell and Figure S9l shows the steady-state photocurrents of 180 s at bias voltages of 0.94 V at the maximum power output.

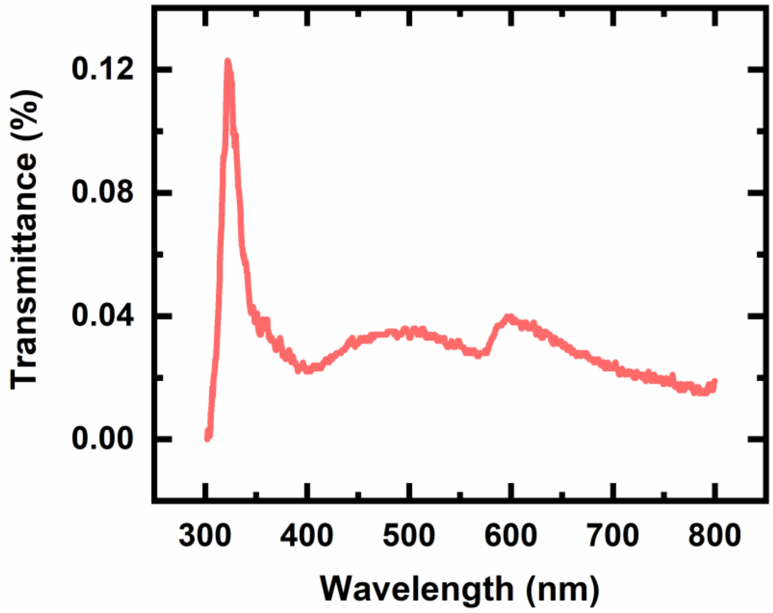

Supplementary Figure 10. The transmittance spectra of solar cell with structure of ITO/SnO_2/$CsFAPbI_3$/Spiro/Au/ITO/Au under 1-Sun illumination.

The memristor is directly stacked onto the solar cell to form self-powered striate cortex which is then attached to hemispherical shaped substrate for the next-stage characterization. Therefore, the self-

powered memristor is inverted on the hemispherical shaped substrate with solar cell on the top of memristor to receive the optical stimuli for pattern recognition (as illustrated in Figure 4c). Sequentially, the solar cell would receive the optical illumination before memristor. By performing the UV-visible absorption spectrum of solar cell with the structure of ITO/SnO_2/$CsFAPbI_3$/Spiro/Au/ITO/Au, we confirm that the maximum transmittance of the solar cell is only 0.12% at the 322 nm, implying that almost negligible light could get through the solar cell to reach and influence the memristor.

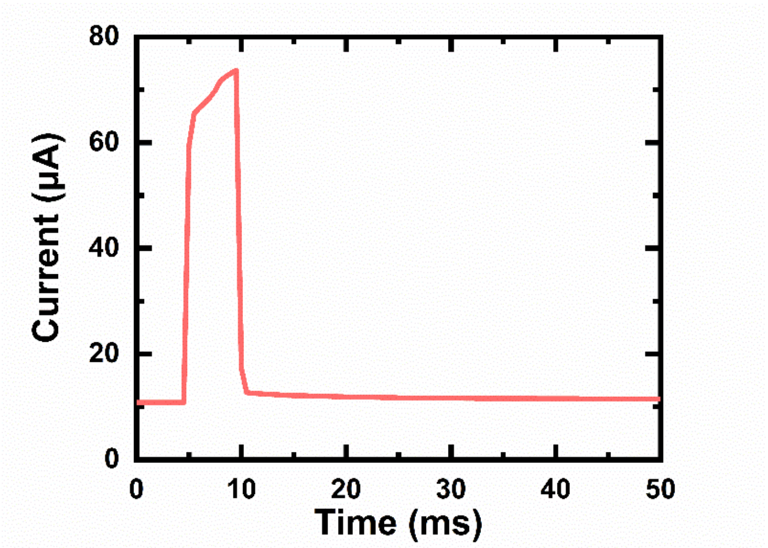

Supplementary Figure 11. The current response of memristor under external voltage pulse with amplitude of 0.5 V and duration of 5 ms.

Regarding compatibility of photovoltaic cells and memristors in terms of power, we performed pulse experiment on the memristor by application of single pulse with amplitude of 0.5 V and duration of 5 ms. The power consumption of single memristor per operation is estimated as $P_{\text{potentiation}} = V \times I \times t =$ 0.5 V $\times$ 70 μA $\times$ 5ms=175 nJ. In addition, more detailed parameters about solar cell used in this work under 1-Sun illumination are provided in Table S3. The solar cell exhibits max power of 0.43 mW, the V_{oc} of 1.1 V, and J_{sc} of 23.7mA/cm^2, indicating that the solar cell could generate enough power to drive the memristor.

Supplementary Table 3. The detailed parameters of the solar cell employed in this work.

Parameter	Area	I_{sc}	V_{oc}	P_{max}	I_{max}	V_{max}	Efficiency	Fill Factor	J_{sc}	J_{max}
Value	0.02cm^2	0.47mA	1.1V	0.43mW	0.45mA	0.95V	21.6%	82.6%	23.7 mA/cm^2	22.7 mA/cm^2

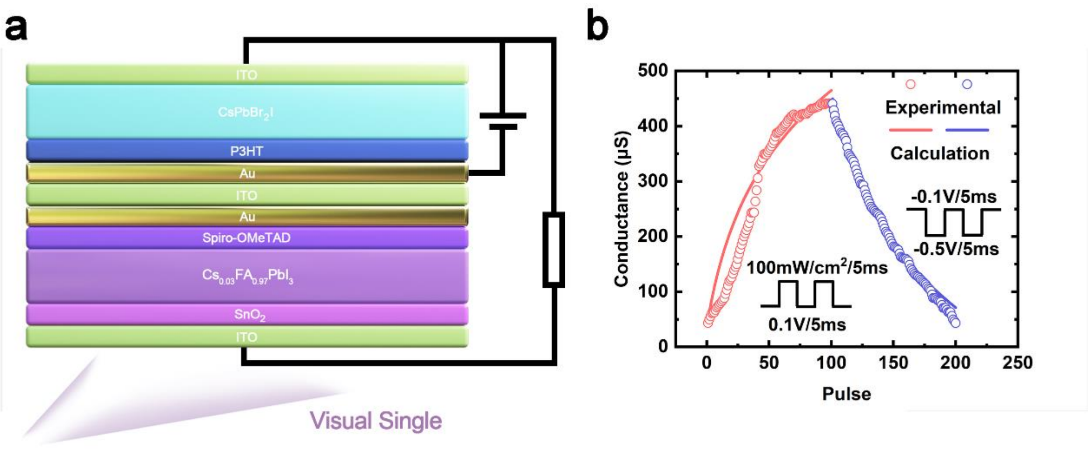

Supplementary Figure 12. The test system and the optoelectronic characteristic of integrated artificial visual cortex.

Figure S12a shows the test system of integrated artificial visual cortex. The artificial visual cortex including a tandem structure of one solar cell and one memristor, a ITO layer is employed as recombination layer to completely protects the underlying solar cell from any solvent damage during the fabrication of memristor. A series resistor (5000 Ω) is used in experimental circuit to divide the high photovoltage. Figure S12b shows the optoelectronic characteristic result, the 100 optical pulses and the following 100 electrical pulses are used to potentiate and depress artificial visual cortex. The NL fitting has also been carried out to demonstrate the great weight modulation phenomenon, the fitting results are summarized in Table S4.

Supplementary Table 4. The fitting results of NL from pulse experimental data.

G_{min}	G_{max}	LTP		LTD	
		α_p	β_p	α_d	β_d
50 μS	450 μS	13 μS	1.9	10 μS	1.8

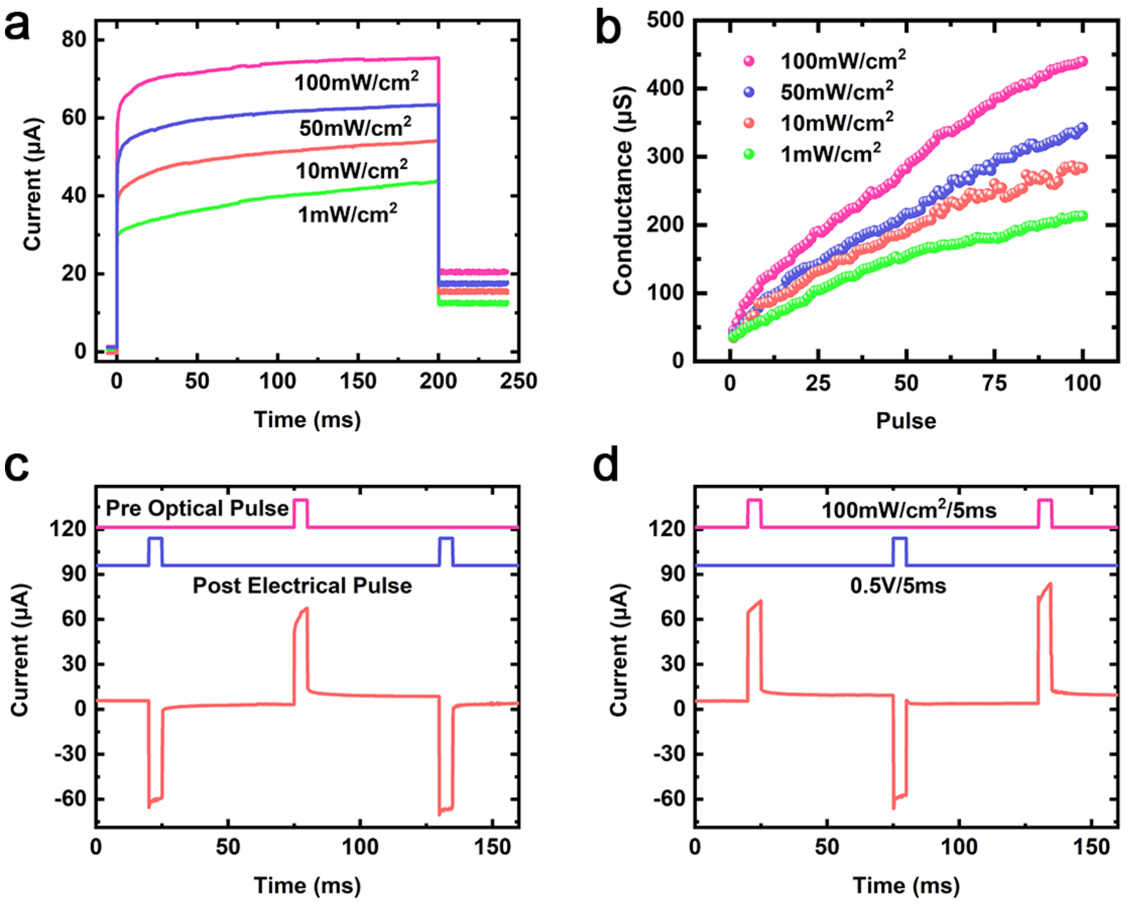

Supplementary Figure 13. a, The current response as the function of 100 mW/cm^2, 50 mW/cm^2, 10 mW/cm^2 and 1 mW/cm^2. **b,** The potentiation behavior as the function of 100 mW/cm^2, 50 mW/cm^2, 10 mW/cm^2 and 1 mW/cm^2, and all the pulse width is set to 5ms. **c,** The current response of 'post-pre-post' pulse sequence, where the pre-synapse and post-synapse are triggered by optical and electrical pulses, respectively. **d,** The current response of 'pre-post-pre' pulse sequence, where the pre-synapse and post-synapse are triggered by optical and electrical pulses, respectively.

As shown in Figure S13a, the current response increases more significantly with the increase of

light intensity, indicating that the conductance of memristor can be modulated by different light intensities. In addition, the higher light intensity could induce larger photovoltaic effect of solar cell to drive memristor to higher conductance states, corresponding to spike amplitude-based synaptic plasticity (Figure S13b). The triplet-STDP learning rule are further implemented in the self-powered memristor to illustrate two typical triplets with 'post-pre-post' and 'pre-post-pre' sequences, as shown in Figure S13c and S13d. The presynaptic optical pulse and postsynaptic electrical pulse are fixed to 100 mW/cm^2 and 0.5 V, and the interval time is fixed to 0.5 ms. The synapse weight change in triplet-STDP can be qualitatively divided to the LTP and LTD behaviors as function of interval times.

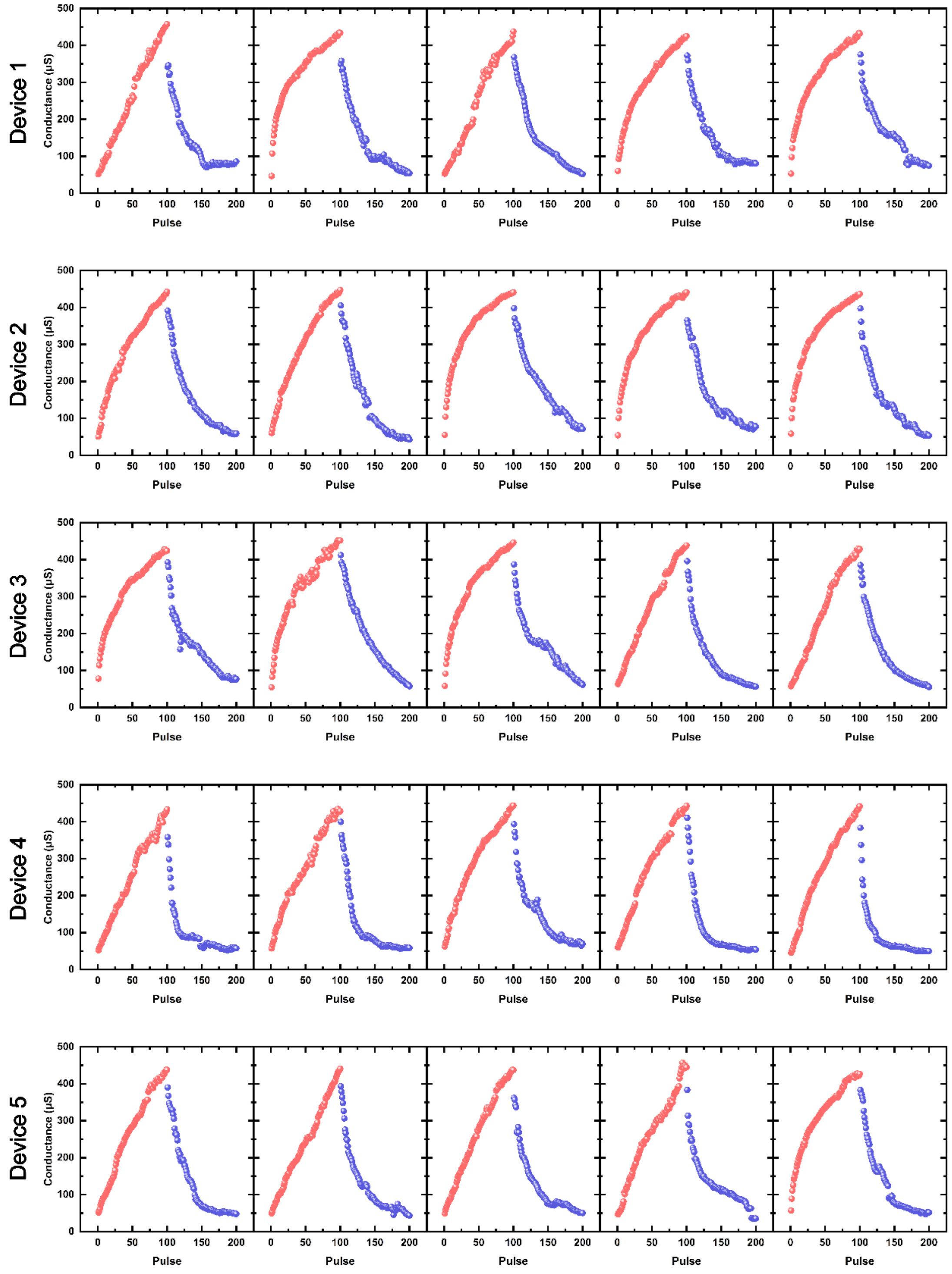

Supplementary Figure 14. The potentiation and depression characteristics of the randomly selected device 1-5.

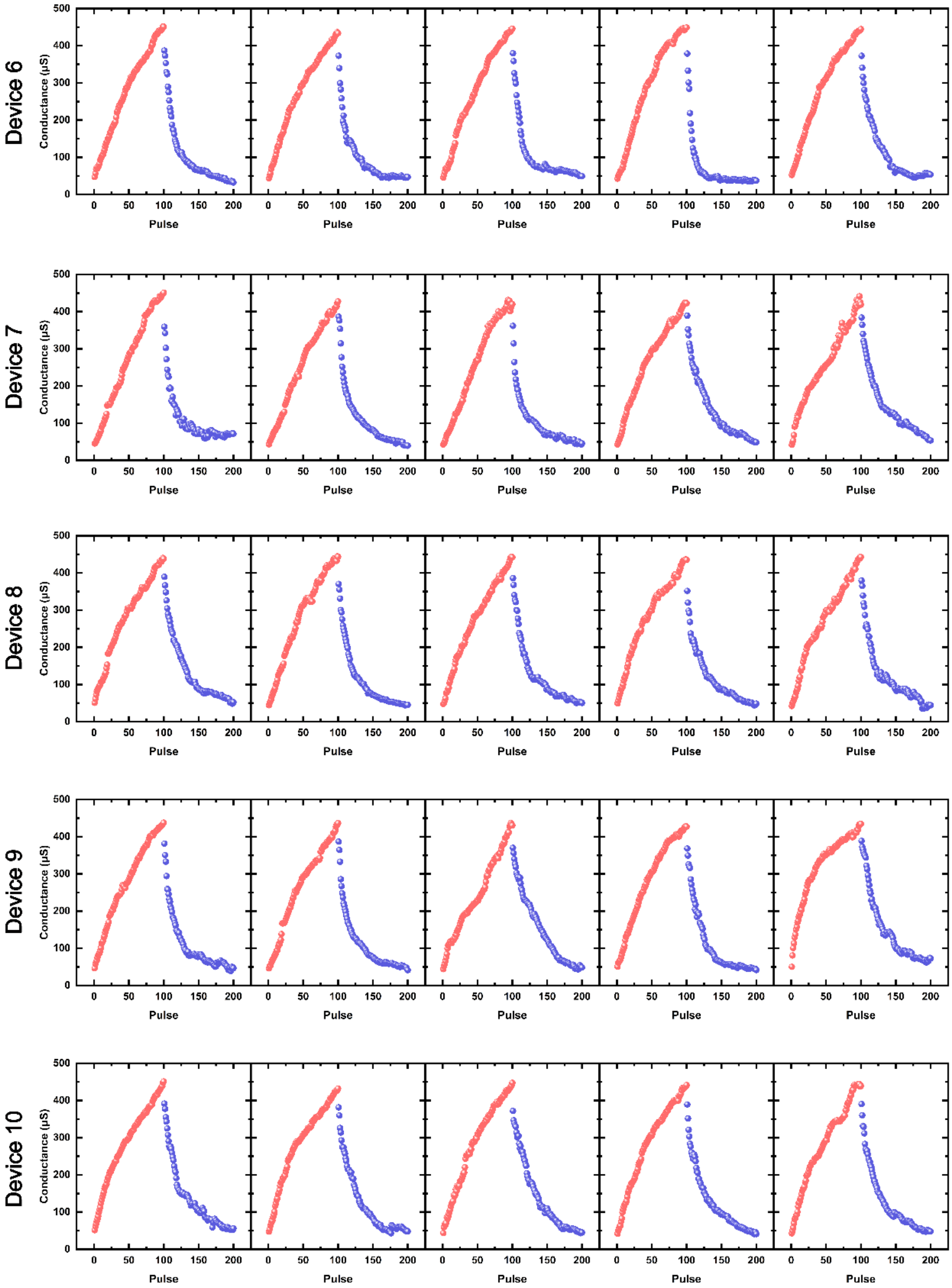

Supplementary Figure 15. The potentiation and depression characteristics of the randomly selected device 6-10.

In order to investigate the uniformity of the artificial striate cortex, we performed 50 pulse experiments on 10 randomly selected devices on the bended array. The potentiation and depression process are triggered by consecutively 100 optical pulses with intensity of 100 mW/cm^2 and duration of

5 ms and consecutively 100 electrical pulses with amplitude of 0.5 V and duration of 5 ms, respectively, all the optical pulses are penetrated from the top direction of hemispherical artificial striate cortex. It is clearly that the device can be programmed to multi states, and the variabilities of G_{max} and G_{min} , described as σ/μ (σ is the standard deviation and μ is the mean value), are calculated to be 0.018 and 0.164, respectively. In addition, the nonlinearity variabilities of LTP and LTD are only 0.431 and 0.046, indicating a great symmetricity of states modulation.

Supplementary Table 5. The mean values, standard deviations and variations of HRS, LRS and nonlinearity in the pulse experiments.

Parameter	G_{max}	G_{min}	α_p	β_p	α_d	β_d
Mean Value	439.77 μS	47.47 μS	10.72 μS	1.59	46.72 μS	3.88
Standard Deviation	7.97 μS	7.78 μS	5.65 μS	0.69	6.43 μS	0.18
Variation	0.018	0.164	0.527	0.434	0.138	0.046

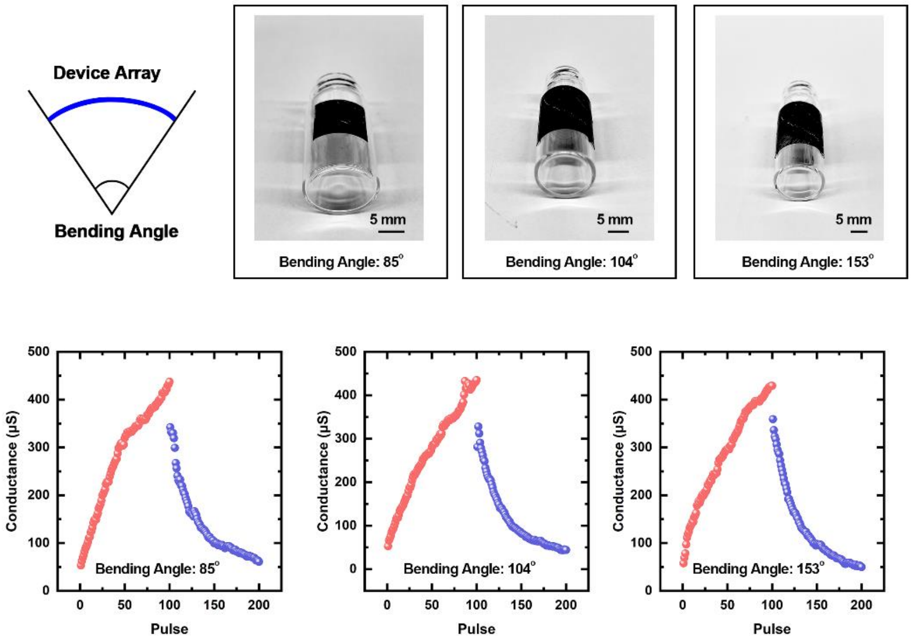

Supplementary Figure 16. Typical potentiation and depression with respect to the different curvatures of the artificial striate cortex. The optical images of the memristor with different bending angles are shown in the upper panel.

By application of continuous 100 optical pulses and 100 electrical pulses, the corresponding potentiation and depression phenomenon as the function of bending angles was provided in Figure S16. The device exhibits stable LTP and LTD performance with bending angles ranging from 85° to 153°, implying its high flexibility.

STDP

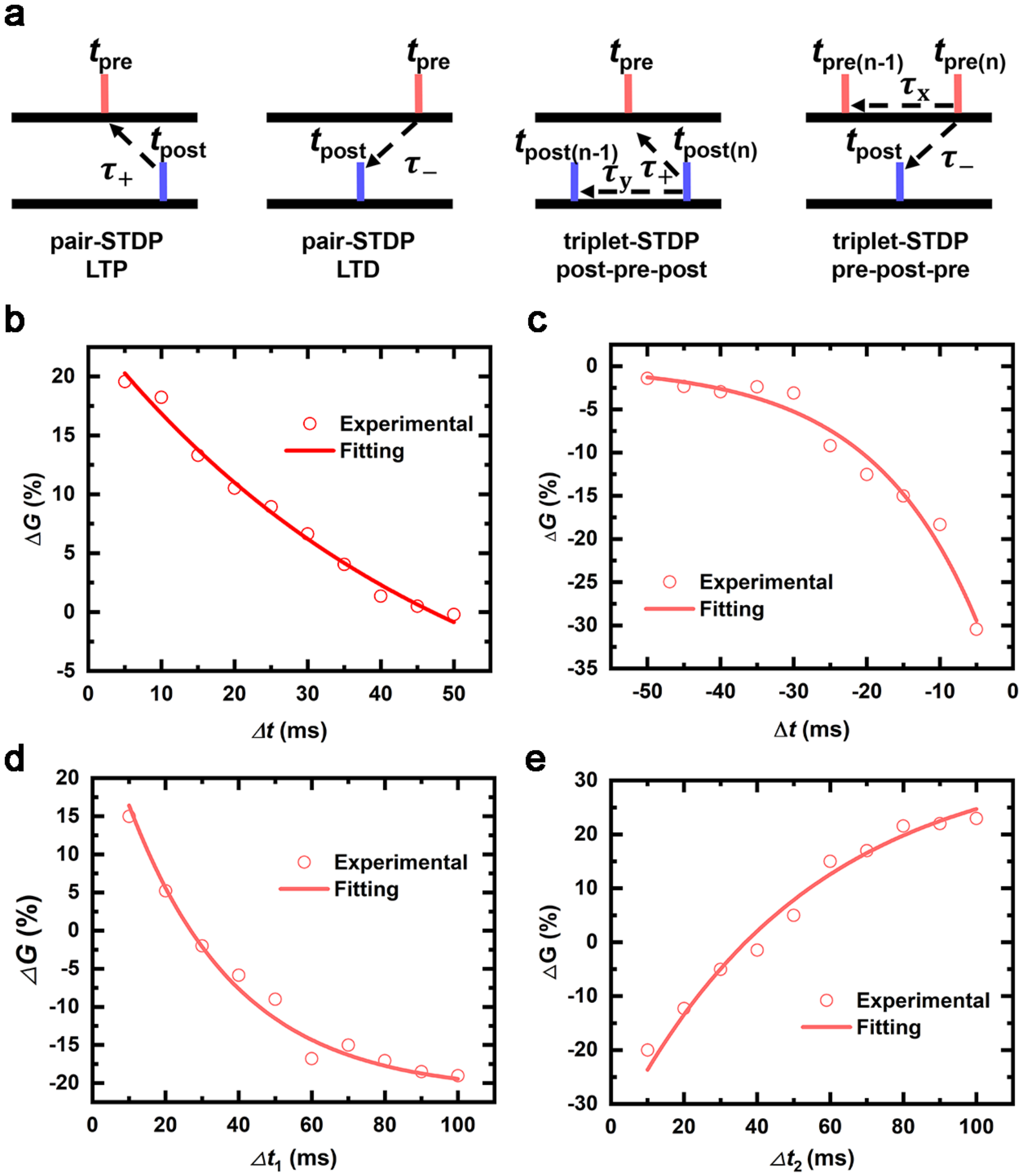

Supplementary Figure 17. Experimental and fitting results of STDP learning rule. a, Schematic illustration of the pair-STDP (LTP and LTD) and triplet-STDP (post-pre-post and pre-post-pre) learning rules. The symmetrical signals are all fixed to 0.5V/5ms, only the interval is the variable in the experiment. **b-c,** Parameter fitting in LTP and LTD of pair-STDP. **d-e,** Parameter fitting in post-pre-post and pre-post-pre of triplet-STDP.

STDP represents the relationship between synaptic weight changes and pre-/postsynaptic pulses interval which follows the rule of exponential equation. The asymmetric pair-STDP, triggered by one presynaptic pulse and one postsynaptic pulse, can be expressed as:

$$\Delta G = \begin{cases} A_2^{+} exp\left(-\Delta t/\tau_{+}\right) & \Delta t > 0 \\ -A_2^{-} exp\left(-\Delta t/\tau_{-}\right) & \Delta t < 0 \end{cases} \tag{2}$$

where the $\Delta t = t_{\text{post}} - t_{\text{pre}}$ is the time interval between postsynaptic and presynaptic pulses, A_2^{+}/A_2^{-} and τ_{+}/τ_{-} are the relevant amplitude and time constants for the potentiation/depression of pair-STDP.

Furthermore, the synaptic weight changes as a function of three synaptic pulses can be named as triplet-STDP. Based on the sequence of presynaptic and postsynaptic pulses, it usually contains two items: pre-post-pre and post-pre-post. Its mathematical representation was approximated by:

$$\Delta G = \begin{cases} exp\left(\frac{-\Delta t}{\tau_+}\right)\left(A_2^+ + A_3^+ exp\left(-\Delta t_1/\tau_y\right)\right) \\ -exp\left(\frac{-\Delta t}{\tau_-}\right)\left(A_2^- + A_3^- exp\left(-\Delta t_2/\tau_x\right)\right) \end{cases} \quad (3)$$

where the $\Delta t_1 = t_{post(n+1)} - t_{post(n)}$ and $\Delta t_2 = t_{pre(n+1)} - t_{pre(n)}$ are the time difference between pre- and postsynaptic pulses. A_3^+ and A_3^- are relevant amplitude, τ_y and τ_x are time constants of the potentiation and depression in triplet-STDP learning rule.

All the Fitting results and the employed data in orientation selectivity simulation are shown in Table S6.

Supplementary Table 6. Fitting results of pair-/triplet-STDP and the parameters used in simulation.

Parameter	A_2^+	A_2^-	τ_+	τ_-	A_3^+	A_3^-	τ_x	τ_y
Experimental	0.39	0.42	48.86 ms	14.56 ms	0.53	0.71	18.70 ms	22.64 ms
Simulation	0	0.42	48.86 ms	14.56 ms	0.53	0	18.70 ms	22.64 ms

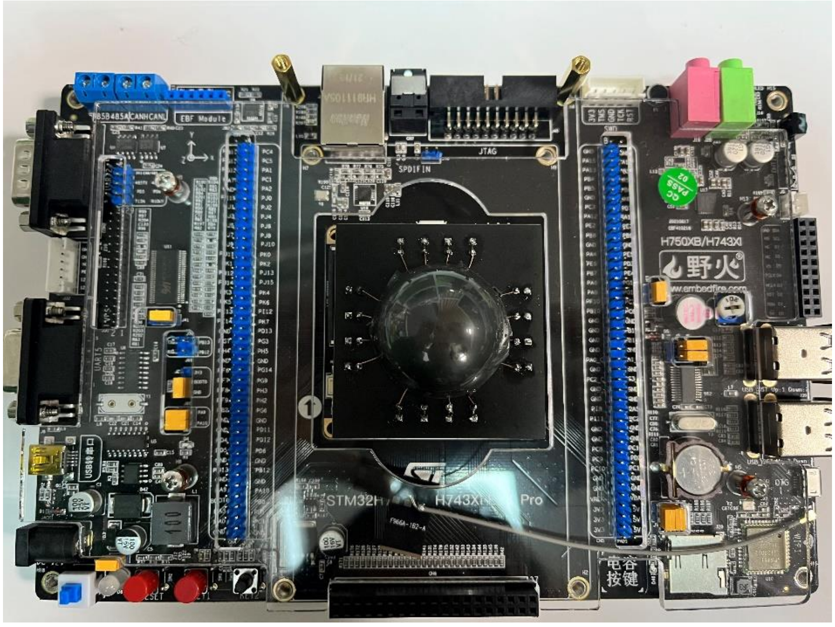

Supplementary Figure 18. The printed circuit board (PCB) of artificial visual cortex.

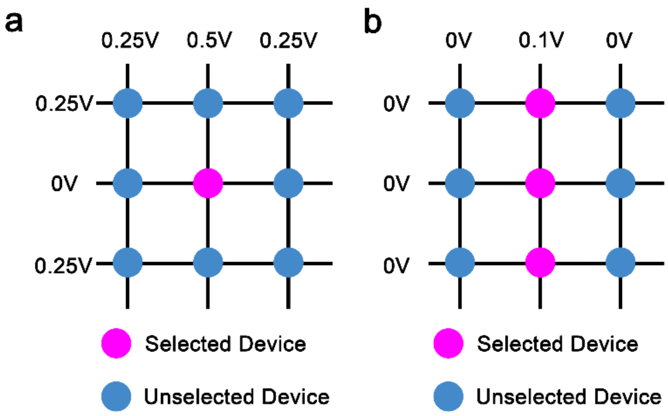

Supplementary Figure 19. a, Weight updating method. **b,** The readout method.

The sneak path problem was avoided by employing programming-inhibit operation in our work. As shown in Figure S19a, the bit line of the selected memristor was applied a programming pulse (0.5V/5ms) and the word line was set to 0 V. In the meantime, all the bit lines and word lines of unselected memristors were applied an inhibit pulses (0.25V/10ms) to prevent the programming disturbance. The application of inhibiting pulses reduces the potential drop between the word line and bit line of unselected memristor which can protect the unselected memristor from state changes. The array states are determined by the following readout process, the selected bit line was set to readout voltage of 0.1V and the other bit lines were kept to 0V, the current were readout from three word lines instantaneously. (Figure S19b)

Simplification Schemes in BCM Learning Rule

In the fitting process of BCM learning rule, several simplifications have been carried out to emulate the BCM features. As mentioned in original manuscript, the BCM learning rule can be described based on the All-to-All framework as $dG/dt = \phi(\rho_y, \theta)\rho_x = \left(-A_2^- \tau_- \rho_y - A_3^- \tau_- \tau_x \rho_x \rho_y + A_2^+ \tau_+ \rho_y + A_3^+ \tau_+ \tau_y \rho_y^2\right)\rho_x$. The first simplification is to view A_3^- as zero. For the requirement of mathematic fitting, the $\phi(\rho_y, \theta) = -A_2^- \tau_- \rho_y - A_3^- \tau_- \tau_x \rho_x \rho_y + A_2^+ \tau_+ \rho_y + A_3^+ \tau_+ \tau_y \rho_y^2$ is designed as scalar function to emulate the essential feature of BCM learning rule: the high post-synaptic firing frequency corresponding to synaptic potentiation ($(\rho_y > \theta, \theta) > 0$), the low post-synaptic firing frequency corresponding to synaptic depression ($(\rho_y < \theta, \theta) < 0$) and the synapse maintaining its state when post-synapse doesn't fire ($(0, \theta) = 0$). According to the biological experimental results, the BCM learning rule for synaptic modification in biological system obey a zero-axial quadratic polynomial with the argument of ρ_y so that setting A_3^- to zero is on-demand. In addition, the A_3^- is much lower than A_2^- ($A_3^- \ll A_2^-$) in biological system which means the effect of the triplet depression term is almost negligible compared with the depression induced by paired spikes. Hence, this simplification will not affect the rationality of emulation of BCM learning in biological system.

Even the previous simplification of $\phi(\rho_y, \theta)$ is capable of emulating the first feature of BCM learning rule, another simplification is still required to mimic the second feature, namely, the sliding threshold. The average value of second power of postsynaptic firing rate $\langle \rho_y^2 \rangle$, which reflects previous history of memristor, is introduced to describe the sliding feature of threshold. The first order term of $\phi(\rho_y, \theta)$ is selected and redefined as $A_2^- \to A_2^- \langle \rho_y^2 \rangle / \rho_0^2$ and $A_2^+ \to A_2^+ \langle \rho_y^2 \rangle / \rho_0^2$, while the second order term is kept unchanged. In conclusion, these simplifications allow us to implement a rational BCM model with relatively less parameters.

Orientation Selectivity Simulation

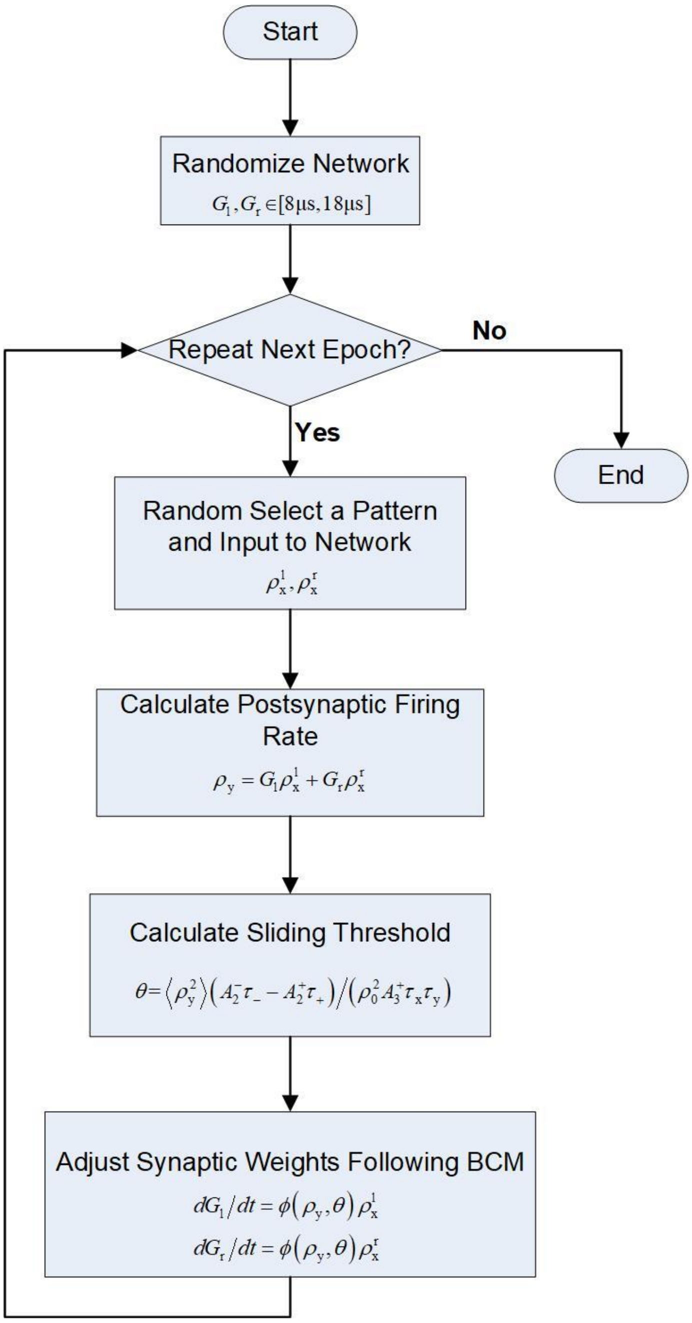

Supplementary Figure 20. The flow chart of the orientation selectivity simulation.

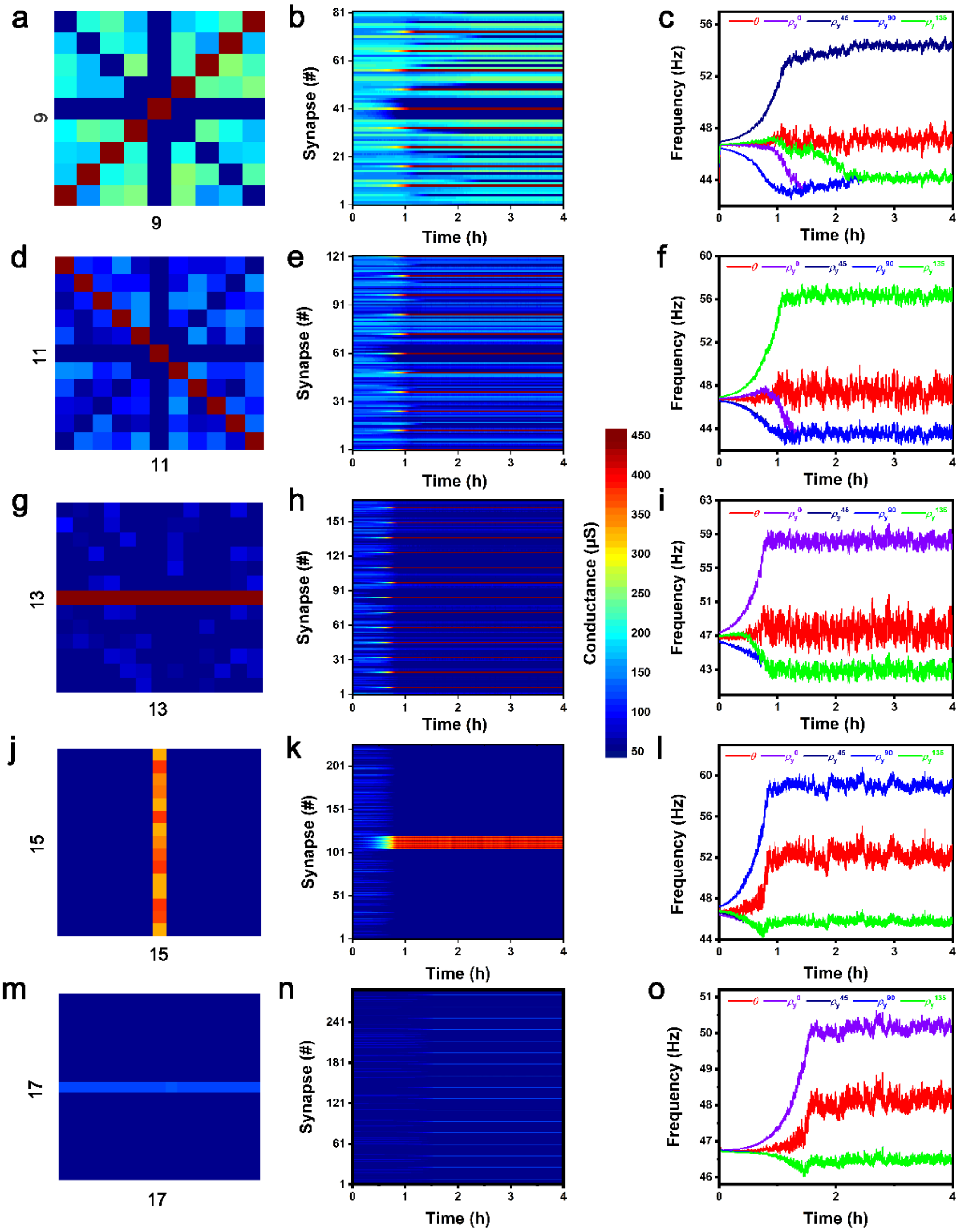

Supplementary Figure 21. a, The finial color map of 9×9 cortical synapse array in normally rearing condition of orientation selectivity simulation. **b,** The evolution of synaptic weights as a function of simulation time. **c,** The evolution of postsynaptic firing frequencies and network threshold as a function of simulation time. **d-o,** The simulation results of 11×11, 13×13, 15×15 and 17×17 cortical synapse arrays.

In order to clarify the influence of array size on the orientation selectivity, we built five arrays with different sizes (9 by 9, 11 by 11, 13 by 13, 15 by 15 and 17 by 17) for orientation selectivity simulation, and all of them can be trained successfully that one orientation can win the network and keep the winner position in the following training epochs. In addition, the orientation selectivity can be evaluated by:

$$\text{Orientation Selectivity} = 1 - \frac{\text{mean}(\rho_y^0, \rho_y^{45}, \rho_y^{90}, \rho_y^{135}) - \min(\rho_y^0, \rho_y^{45}, \rho_y^{90}, \rho_y^{135})}{\max(\rho_y^0, \rho_y^{45}, \rho_y^{90}, \rho_y^{135}) - \min(\rho_y^0, \rho_y^{45}, \rho_y^{90}, \rho_y^{135})} \quad (4)$$

where the ρ_y^0, ρ_y^{45}, ρ_y^{90} and ρ_y^{135} are the response frequency for the orientation of 0°, 45 °, 90 ° and 135 °, respectively. The simulation results were summarized in Table S7 and Figure S21. It is clear that the orientation selectivity cannot be improved by increasing array size. In our work, the orientation bar is kind of small-sample pattern with limited number of pixels and pattern features, and a 9 by 9 array can effectively perform feature extraction and classification during training and test processes for orientation selectivity task. Larger array size may induce the high energy consumption in this scenario.

Supplementary Table 7. The orientation selectivity based on different array sizes.

Array Size	9 × 9	11 × 11	13 × 13	15 × 15	17 × 17
Orientation Selectivity (%)	74.99	75.00	74.99	75.00	74.87

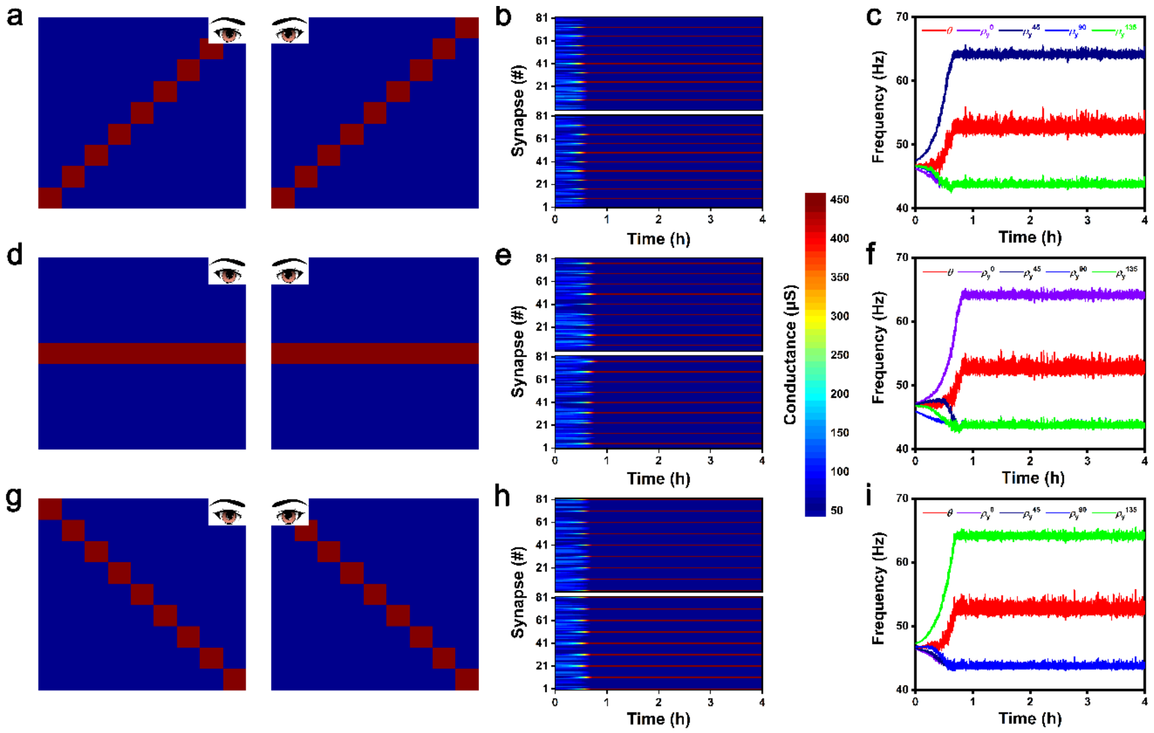

Supplementary Figure 22. a, The finial color maps of 9×9 cortical synapse arrays with the 45° as the winner of orientation selectivity simulation, including two arrays for left and right eyes. **b,** The evolution of synaptic weights as the function of simulation time, where the upper and lower behalf are corresponding to left and right eyes. **c,** The evolution of postsynaptic firing frequencies and network threshold as a function of simulation time. It is clearly that only ρ_y^{45} is larger than firing threshold which means the vertical is the selected orientation. **d-i,** Similarly, the simulation results with the winner orientation of 0 ° and 135 °.

In addition, we performed orientation selectivity simulation for different orientation of 45°, 0° and 135°, as shown in Figure S22. All of them displays similar results with the orientation of 90° in the manuscript. And 100 repeat simulations are performed to count the probability of different orientation selectivity, as summarized in Table S8. The statistical result shows that all the orientation can be learned with similar probability.

Supplementary Table 8. The winner time of four orientations in 100 simulations.

Orientation	0 °	45 °	90 °	135 °
Time	23	26	26	25
